# Altered Middle Ear Microbiome in Children With Chronic Otitis Media With Effusion and Respiratory Illnesses

**DOI:** 10.3389/fcimb.2019.00339

**Published:** 2019-10-04

**Authors:** Allison R. Kolbe, Eduardo Castro-Nallar, Diego Preciado, Marcos Pérez-Losada

**Affiliations:** ^1^Department of Biostatistics and Bioinformatics, Milken Institute School of Public Health, Computational Biology Institute, The George Washington University, Washington, DC, United States; ^2^Facultad de Ciencias de la Vida, Center for Bioinformatics and Integrative Biology, Universidad Andrés Bello, Santiago, Chile; ^3^Division of Pediatric Otolaryngology, Sheikh Zayed Institute, Children's National Health System, Washington, DC, United States; ^4^CIBIO-InBIO, Centro de Investigação em Biodiversidade e Recursos Genéticos, Universidade Do Porto, Vairão, Portugal

**Keywords:** otitis media, asthma, bronchiolitis, middle ear microbiome, amplicon sequence variants

## Abstract

Chronic otitis media with effusion (COME) is a common childhood disease characterized by an accumulation of fluid behind the eardrum. COME often requires surgical intervention and can also lead to significant hearing loss and subsequent learning disabilities. Recent characterization of the middle ear fluid (MEF) microbiome in pediatric patients has led to an improved understanding of the microbiota present in the middle ear during COME. However, it is not currently known how the MEF microbiome might vary due to other conditions, particularly respiratory disorders. Here, we apply an amplicon sequence variant (ASV) pipeline to MEF 16S rRNA high-throughput sequencing data from 50 children with COME (ages 3–176 months) undergoing tube placement. We achieve a more detailed taxonomic resolution than previously reported, including species and genus level resolution. Additionally, we provide the first report of the functional roles of the MEF microbiome and demonstrate that despite high taxonomic diversity, the functional capacity of the MEF microbiome remains uniform between patients. Furthermore, we analyze microbiome differences between children with COME with and without a history of lower airway disease (i.e., asthma or bronchiolitis). The MEF microbiome was less diverse in participants with lower airway disease than in patients without, and phylogenetic β-diversity (weighted UniFrac) was significantly different based on lower airway disease status. Differential abundance between patients with lower airway disease and those without was observed for the genera *Haemophilus, Moraxella, Staphylococcus, Alloiococcus*, and *Turicella*. These findings support previous suggestions of a link between COME and respiratory illnesses and emphasize the need for future study of the middle ear and respiratory tract microbiomes in diseases such as asthma and bronchiolitis.

## Introduction

Chronic otitis media with effusion (COME) is characterized by an accumulation of fluid behind the eardrum which persists for 3 months or more, typically without signs of inflammation (Minovi and Dazert, 2014). COME is a leading cause of hearing loss in children, especially in developing countries (Monasta et al., 2012), and can result in learning disabilities and educational problems (Williams and Jacobs, 2009). Approximately 80% of children worldwide will have experienced an episode of COME by age 10 (Minovi and Dazert, 2014).

More than half of COME cases are preceded by acute otitis media (AOM), which are most commonly caused by bacterial infection with *Streptococcus pneumoniae, Moraxella catarrhalis*, and *Haemophilus influenzae* (Minovi and Dazert, 2014; Qureishi et al., 2014). However, the role of these pathogens or other components of the middle ear microbiome in COME is less well-understood. All three of these pathogens have been identified in middle ear fluid (MEF), as well as *Alloiococcus otitis, Turicella otitidis*, and *Staphylococcus* sp. (Harimaya et al., 2006; Guvenc et al., 2010; Jervis-Bardy et al., 2015; Chan et al., 2016; Lappan et al., 2018). Interestingly, several of these same species have been linked to the development of asthma and the asthma microbiome, particularly *M. catarrhalis* and *H. influenzae* (Bisgaard et al., 2007; Castro-Nallar et al., 2015; Pérez-Losada et al., 2015). However, the relationship between asthma and the middle ear microbiome, particularly in the context of COME, is not known.

Several studies have observed a relationship between otitis media (OM) and respiratory illnesses such as asthma and bronchiolitis. Bronchiolitis is a lung infection that most commonly affects young children at the same age as typical AOM incidence and is associated with airway inflammation and congestion. Asthma is a chronic condition characterized by airway inflammation and increased mucus production, and can result in shortness of breath, coughing, and wheezing, typically at an older age. Bacterial AOM is a common complication associated with bronchiolitis (Andrade et al., 1998; Gomaa et al., 2012). Similarly, large-scale studies in Germany and Mexico have observed that AOM episodes in infancy increases the risk of asthma development later in life (Eldeirawi et al., 2010; MacIntyre et al., 2010). Similar relationships have been observed between COME and asthma. A retrospective study of tube placement patients with COME found a significantly higher rate of asthma diagnosis at follow-up compared to children of the same age, as well as different clinical presentations of COME including mucoid effusion and a higher rate of multiple tube placements (Gamble et al., 1992). Interestingly, COME has also been associated with other atopic diseases such as allergic rhinitis (Alles et al., 2001; Luong and Roland, 2008; Kreiner-Moller et al., 2012). Although the directionality of this relationship is unclear, some have hypothesized that chronic inflammation due to asthma or other atopic diseases can reduce eustachian tube opening and impair mucociliary function (Alles et al., 2001; MacIntyre and Heinrich, 2012). Gamble et al. (1992) suggested that COME in asthmatics is not an isolated disease, but rather the result of atopic disease affecting the mucociliary system of the entire respiratory tract. While the link between bronchiolitis and COME has not been explored as extensively, it is possible that inflammation due to bronchiolitis could have a similar effect. At present, however, the nature of the relationship between COME and respiratory illnesses is poorly understood.

Microbiome dysbiosis has been well-characterized in the respiratory tract associated with asthma and bronchiolitis (Hilty et al., 2010; Huang et al., 2011; Marri et al., 2013; Castro-Nallar et al., 2015; Pérez-Losada et al., 2015; Hasegawa et al., 2016; Mansbach et al., 2016; Kozik and Huang, 2018), but it is not currently known whether this dysbiosis extends to the middle ear, particularly when associated with COME. In this study, we sought to characterize the MEF microbiome associated with COME in patients with and without asthma or bronchiolitis. To accomplish this goal, we re-analyzed the 16S rRNA high-throughput sequencing data previously published by Krueger et al. (2017), incorporating new metadata relating to lower airway disease diagnosis as well as analyzing the metabolic function of the microbial communities. Furthermore, we built upon the previous study by utilizing an amplicon sequence variant (ASV) approach which enhances taxonomic classification and reproducibility (Callahan et al., 2017). This study provides new insight into the MEF microbiome in COME, as well as MEF microbiome changes associated with asthma or bronchiolitis.

## Materials and Methods

Sample collection and DNA sequencing methodology for this study were previously described (Krueger et al., 2017). Briefly, middle ear effusion samples were collected from 50 children with chronic otitis media undergoing myringotomy with tympanostomy tube placement at Children's National Health System in Washington, D.C. The cohort ranged from 3 to 176 months of age, with 34 boys and 16 girls. Out of the 50 children, nearly three-fourths suffered from significant hearing loss (36/50). None of these children were treated with antibiotics for 2 weeks prior to sampling. Other (non-antibiotic) medication use was not recorded for this study. Thirteen of the children were diagnosed with lower airway disease such as asthma or bronchiolitis. To fit the categorization of being positive for these, the children needed to meet any of the following criteria: (1) history of pulmonary physician-diagnosed asthma; (2) documented chronic wheezing being treated with a daily respiratory inhaler; or (3) PCR (+) for rhinovirus bronchiolitis diagnosis. Although asthma and bronchiolitis are considered different respiratory illnesses, there often is a spectrum of disease over time and they cannot be reliably distinguished in young children. Therefore, we evaluated them together as lower airway disease in this study.

DNA purification from MEF was performed using the QiaAmp mini kit (Qiagen) and extracted following the MiSeq SOP protocol described in Kozich et al. (2013). The V4 region of the 16S rRNA gene was amplified and libraries were sequenced using the Illumina MiSeq at University of Michigan. Negative controls did not amplify, indicating that bacterial DNA was not present in the reagents, and therefore were not sequenced.

Raw fastq files from Krueger et al. (2017) were re-processed using dada2 version 1.12 (Callahan et al., 2016). This pipeline offers improved taxonomic resolution and reproducibility compared to OTU-based methods (Callahan et al., 2017). Reads were filtered using standard parameters, with no uncalled bases, maximum of two expected errors, and truncating reads at a quality score of two or less. Forward and reverse reads were truncated after 240 and 225 bases, respectively. The standard dada2 pipeline was then applied to perform amplicon sequence variant (ASV) inference, merge paired reads, and identify chimeras. Singleton ASVs are discarded in the dada2 pipeline (Callahan et al., 2017). Taxonomic assignment was performed against the Silva v132 database (Quast et al., 2013) using the dada2-formatted training files for taxonomy and species-level assignment (Callahan, 2018). ASV sequences were aligned using MAFFT (Katoh and Standley, 2013) and used to build a tree with FastTree (Price et al., 2010). The resulting ASV tables and phylogenetic tree were imported into phyloseq (McMurdie and Holmes, 2013) for further analysis.

Functional analysis of microbial communities was performed with PICRUSt2 (Douglas et al., 2019), following the standard pipeline. First, ASVs were aligned to reference sequences using HHMER (Howard Hughes Medical Institute, 2018) and placed into a reference tree with EPA-NG (Barbera et al., 2019) and GAPPA (Czech and Stamatakis, 2019). Hidden-state prediction was performed using castor (Louca and Doebeli, 2018), and along with the ASV abundance table generated by dada2, used to generate metagenome predictions. Finally, KEGG pathway level predictions were performed with MinPath (Ye and Doak, 2009). Exploratory analysis of functional abundances was performed in STAMP (Parks et al., 2014), and visualized with BURRITO (McNally et al., 2018). For visualization purposes, ASVs with <0.1% relative abundance were removed. To test for significant associations between functional profiles and clinical variables, general linear models implemented using MaAsLin, with significance considered at *q* < 0.25 as recommended by the authors (Morgan et al., 2012).

Alpha diversity indices (ASV richness and Shannon diversity) were calculated on raw counts using the *estimate_richness*() function in phyloseq and plotted with ggplot2 (Wickham, 2016). Significant differences based on asthma/bronchiolitis status were identified using linear models with clinical metadata (Muc5B +/−, Muc5AC +/−, significant hearing loss +/−, gender, over/under 24 months of age, mucoid/serous fluid) from Krueger et al. (2017) as covariables.

Prior to calculating beta diversity, read counts were normalized for with DESeq2 (Love et al., 2014), using the modified geometric mean (“poscounts”) as implemented in the function *estimateSizeFactors*(). Beta diversity was calculated using the weighted and unweighted UniFrac distances implemented in the phyloseq package. Significance was determined by PERMANOVA using the R package vegan (Oksanen et al., 2019) with clinical metadata as covariables.

Differential abundance testing was performed with DESeq2 (Love et al., 2014) on normalized data as described before (McMurdie and Holmes, 2013). Using the negative binomial model implemented in DESeq2 accounts for library size differences and biological variability more appropriately than rarefying or using simple proportions (McMurdie and Holmes, 2013). The same variables and covariables listed above were used in the model formula. Significance was determined at α < 0.05. All analyses were performed in R version 3.6 (R Core Team, 2019).

## Results

### Taxonomic Composition of the Ear Microbiome During Chronic Infection

**Figure 1:**
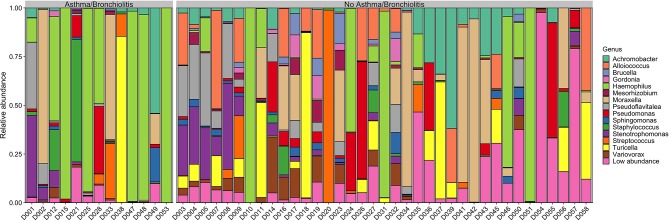
Genus-level relative abundance in patients with or without asthma/bronchiolitis. Genera with mean relative abundance <1% across all samples are plotted as “Low abundance”.

On average, 98% of sequences were assigned at the genus level (Figure 1), compared to ~84% in Krueger et al. (2017). These included two previously unidentified genera, *Achromobacter* and *Pseudoflavitalea*. Furthermore, species level resolution was achieved for some ASVs. These included *Turicella otitidis* (6.9 ± 2.2%), *Alloiococcus otitis* (6.0 ± 1.7%), and *Stenotrophomonas maltophilia* (4.6 ± 1.4%), as well as several low abundance (<1%) ASVs (Supplementary Table 1).

The taxonomic composition of the middle ear effusion is quite diverse, with no core microbiome. Although *Haemophilus, Moraxella*, and *Turicella* were the largest genera by mean relative abundance, these genera were only present in approximately half of the studied samples (27/50, 26/50, and 26/50, respectively). *Achromobacter* and *Pseudomonas* were the most prevalent genera, present in 39/50 and 38/50 samples, respectively (Supplementary Table 2).

### Metabolic Function of the Ear Microbiome During Chronic Infection

**Figure 2:**
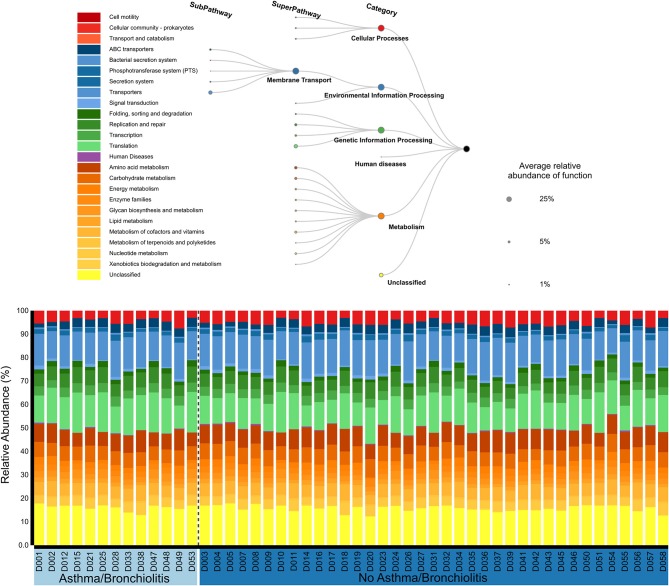
Relative abundances of KEGG functional pathways classified by PICRUSt2 (Douglas et al., 2019) and visualized with BURRITO (McNally et al., 2018).

The functional roles of the middle ear microbiome were primarily classified into four groups: cellular processes (4.7 ± 0.2%), environmental information processing (20.4 ± 0.3%), genetic information processing (24.8 ± 0.5%), and metabolism (33.8 ± 0.3%). Approximately 16% were unclassified. The sub-pathways with the largest relative abundance were Membrane Transport (19.4 ± 0.3%), Translation (13.4 ± 0.4%), Amino acid metabolism (7.6 ± 0.2%), and Carbohydrate Metabolism (6.3 ± 0.1%). Functional profiles were similar across all patients (Figure 2). No significant associations were identified between the functional profiles and clinical variables using MaAsLin or principal component analysis.

### Variation of Ear Microbiome Composition and Function During Chronic Infection

**Figure 3:**
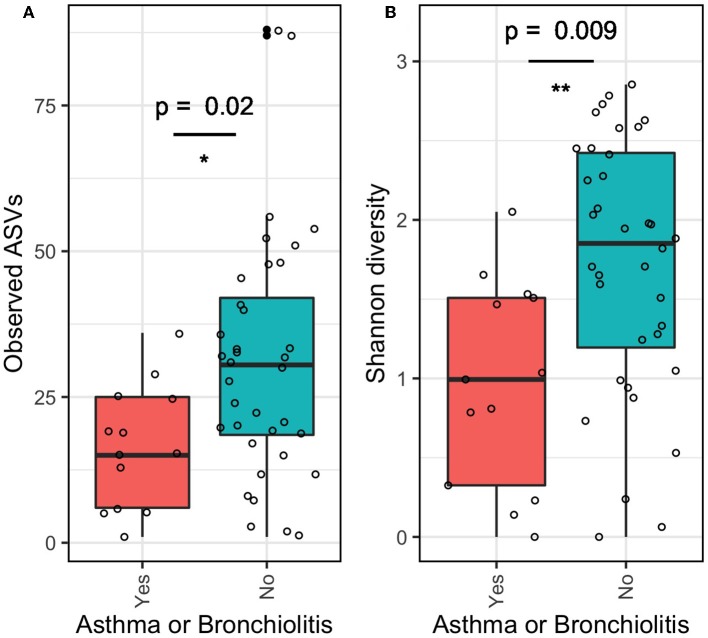
α-diversity of middle ear fluid in patients with asthma or bronchiolitis. Estimates of richness (**A**; number of observed ASVs) and evenness (**B**; Shannon diversity) were significantly lower in patients with asthma or bronchiolitis. *p < 0.05, **p < 0.01.

The α-diversity of the middle ear microbiome estimated by ASV richness and Shannon diversity indices was significantly lower in patients with asthma or bronchiolitis than in patients without (Figure 3; *p* < 0.05). Mean ASV richness in patients with asthma/bronchiolitis was 16.4 compared to 31.1 in patients without; similarly, mean Shannon diversity in patients with asthma/bronchiolitis was 0.96 compared to 1.72 in patients without. α-diversity did not vary significantly between other clinical variables, including age, gender, significant hearing loss, mucoid/serous effusion, and presence of Muc5B and/or Muc5AC.

**Figure 4:**
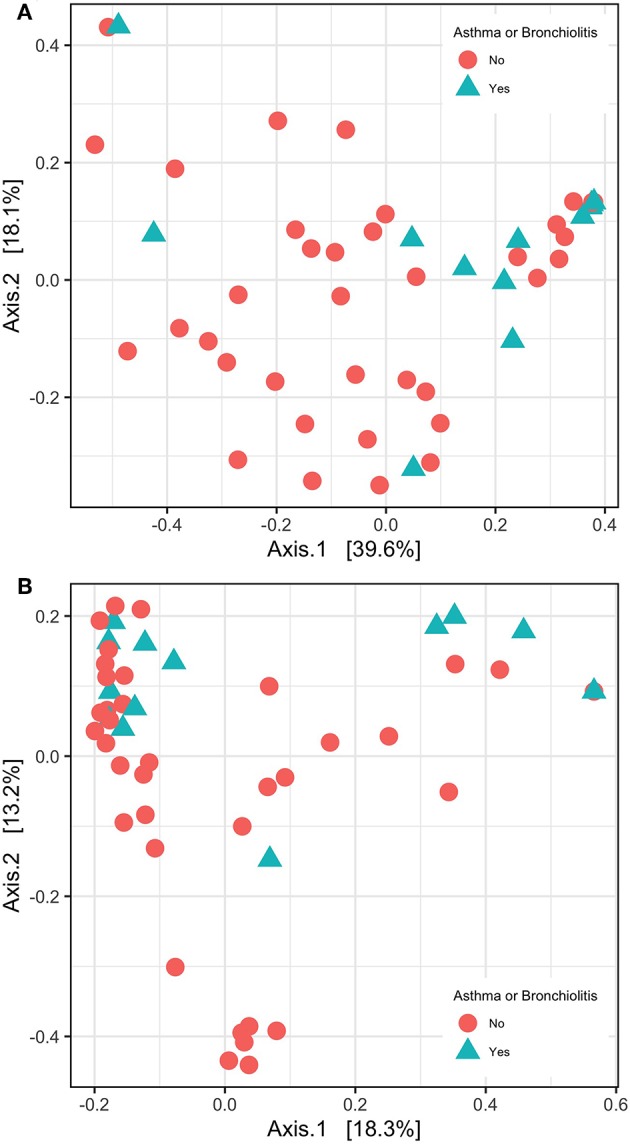
β-diversity shown with principle coordinate analysis of weighted UniFrac distances **(A)** and unweighted UniFrac distance **(B)**.

In contrast, principle coordinate analysis with weighted UniFrac distances and analysis with PERMANOVA indicated significant differences in β-diversity between patients with/without significant hearing loss and asthma/bronchiolitis status (Figure 4A). These variables were not significant in principle coordinate analysis with unweighted UniFrac distances (Figure 4B).

**Figure 5:**
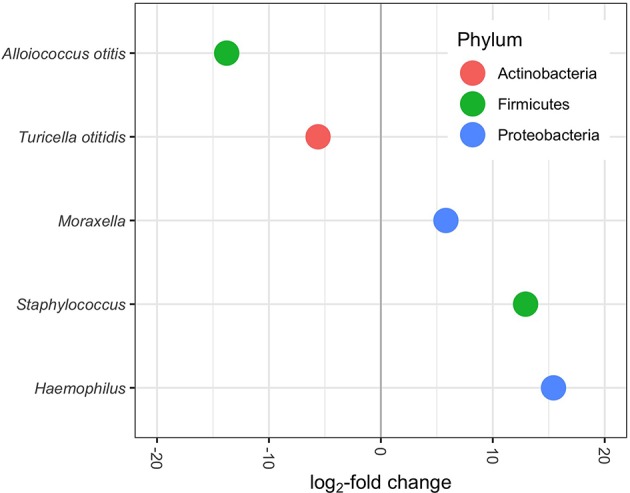
Differentially abundant genera/species (*p-adjusted* < 0.05) in MEF of patients with lower airway disease diagnosis. Positive log_2_-fold change indicates increased abundance in patients with asthma or bronchiolitis; negative log_2_-fold change indicates decreased abundance. Each dot represents one ASV.

Differential abundance testing with DESeq2 identified several ASVs that varied significantly in the relative mean proportions with respect to asthma/bronchiolitis status, after accounting for variation in other clinical and demographic variables. *Haemophilus, Staphylococcus*, and *Moraxella* were significantly higher in children with asthma or bronchiolitis, whereas *Turicella* and *Alloiococcus* were significantly lower (Figure 5).

## Discussion

Chronic otitis media with effusion (COME) is the leading cause of hearing loss among children and affects as many as 80% of children by age 10 (Minovi and Dazert, 2014). Here, we present novel insights from the 16S data presented in Krueger et al. (2017), using amplicon sequence variants (ASVs) instead of OTUs, as well as evaluating links to diagnosed lower airway disease.

Using exact sequence variants instead of OTUs allows for greater precision and reproducibility in taxonomic assignment (Callahan et al., 2017, 2019; Edgar, 2018; Knight et al., 2018; Xue et al., 2018). Previous OTU-based approaches typically functioned by clustering sequences based on a 97% similarity threshold, and then assigning these clusters to reference tree-based OTUs. These approaches did not incorporate sequence quality information or statistical information about the reads into taxonomic assignments, and were therefore losing valuable information (Callahan et al., 2017). Using exact sequence variants improves estimations of diversity and taxonomic predictions, especially for communities which have not been studied extensively (Callahan et al., 2017; Caruso et al., 2019). With the approach implemented in dada2 (Callahan et al., 2016), we were able to achieve species level classifications for multiple ASVs, three of which were present at a mean relative abundance >1%. Of these, *Alloiococcus otitis* and *Turicella otiditis* have been previously identified as typical components of the OM microbiome (Tano et al., 2008; von Graevenitz and Funke, 2014; Lappan et al., 2018). In contrast, *Stenotrophomonas maltophilia* has not been described in many middle ear microbiomes, despite being identified as the source of numerous human diseases including respiratory infections (Brooke, 2012). However, recent work by Kalcioglu et al. (2018) identified *S. maltophilia* in tympanosclerotic plaques isolated from an individual undergoing surgery due to COME. Therefore, *S. maltophilia* may be found in a subset of COME patients, particularly those who have developed tympanosclerosis. Interestingly, *S. maltophilia* was not found in cholesteatomas in the same study, indicating it may serve a specialized role (Kalcioglu et al., 2018).

Furthermore, OTUs that had been previously classified at the family level only were able to be defined at the genus level now using dada2. These were *Achromobacter* (relative abundance: 6.6%, family: Alcaligenaceae) and *Pseudoflavitalea* (relative abundance: 5.3%, family: Chitinophagaceae). *Pseudoflavitalea* is a newly described genus that currently has only been isolated from soil samples (Kim et al., 2016); its potential role in human disease is unknown. On the other hand, *Achromobacter xylosoxidans* was initially isolated from ear discharge from OM patients (Yabuuchi and Ohyama, 1971) and was recently identified in cholesteatomas of multiple individuals with COME (Kalcioglu et al., 2018). Members of the *Achromobacter* genus, specifically *Achromobacter xylosidans*, have been shown to be opportunistic respiratory pathogens, particularly in patients with cystic fibrosis or immune deficiencies (Swenson and Sadikot, 2015). Given that *Achromobacter* was the most prevalent genus identified (present in 39/50 samples), with a mean relative abundance of 6.6%, further work on the role of *Achromobacter* in COME is warranted.

The high inter-patient variation and lack of core microbiome indicates that there is no typical middle ear microbiome associated with COME (Figure 1). In contrast to previous reports that found the middle ear microbiome to be dominated by a single genus, typically from *Alloicoccus, Moraxella, or Haemophilus* (Guvenc et al., 2010; Jervis-Bardy et al., 2015), the middle ear microbiome in this study was highly variable, generally compromising at least two high-abundance genera. This difference may be due to the finer resolution of the ASV pipeline implemented in dada2, and the larger quantity of data retained by not rarefying the counts. Furthermore, even the “typical” agents associated with AOM—namely, *Haemophilus, Moraxella*, and *Streptococcus*—were absent in ~50% of the samples from our COME patients. These results highlight the complexity of the middle ear microbiome associated with COME, which is perhaps expected given that a multitude of risk factors and potential etiologies have been identified for COME.

To our knowledge, we present here the first characterization of microbiome function in the middle ear. Although taxonomic composition varied dramatically between patients, microbiome function was remarkably similar across all patients (Figure 2). This supports the idea that microbial community functions often converge despite different taxonomic compositions (Human Microbiome Project, 2012; Louca et al., 2018). These will include core functions that are essential for microbial life, such as translation (Human Microbiome Project, 2012), which was highly abundant in our analysis. Interestingly, one of the top functions of the middle ear microbial community was “Membrane Transport,” which includes a wide range of genes such as transporters, the phosphotransferase system, and the bacterial secretion system. In our case, the majority of genes were subcategorized in the general category “Transporters.” In previous human microbiome studies, membrane transport has been identified as an abundant component of the healthy laryngeal microbiome (Jette et al., 2016) and associated with gut microbiome dysbiosis in irritable bowel disease and obesity (Greenblum et al., 2012). Further work is necessary to understand if these functions represent unique roles of the microbial community during COME, or if overlapping functions are present in AOM and healthy MEF microbiome.

Microbial diversity varied significantly between COME patients with and without asthma/bronchiolitis (Figures 3–5). Measures of α-diversity indicated that both richness and evenness were lower in COME patients with asthma or bronchiolitis. Although α-diversity of the lower respiratory tract has been shown to be higher (Huang et al., 2011; Marri et al., 2013) or unchanged (Goleva et al., 2013) in asthma patients, Castro-Nallar et al. (2015) showed decreased α-diversity in the nasal cavities of asthma patients compared to healthy patients. Decreased α-diversity has also been observed in microbiome profiles associated with increased risk of bronchiolitis (Hasegawa et al., 2017). Therefore, it is possible that the upper and lower respiratory tracts exhibit distinct trends in asthma patients, with decreased α-diversity in the upper respiratory tract extending to the middle ear and increased α-diversity in the lower respiratory tract. This would lend credence to the theory of a unified airway, which suggests that upper and lower airway disease share a pathophysiologic origin, being induced by a commonality in allergic or non-allergic reproducible mechanisms (Giavina-Bianchi et al., 2016). To this point, it is noteworthy that in patients with an allergic asthmatic etiology, a parallel type of allergic inflammatory response is noted in the middle ear (Nguyen et al., 2004). Moreover, patients with asthma are well-known to present with comorbid conditions which contribute to respiratory symptoms, including allergic rhinitis and chronic rhinosinusitis. Epidemiological and pathophysiological links have been described between these conditions (Massoth et al., 2019). This study's microbiome data builds on this point, suggesting an association between asthma and otitis media. However, due to potential bias between different methodologies, further work using paired samples would be necessary to confirm this hypothesis.

Using the weighted UniFrac distance method, we observed significant differences in β-diversity based on two clinical variables: significant hearing loss and asthma/bronchiolitis diagnosis (Figure 4). The association with significant hearing loss was previously observed in Krueger et al. (2017) and was also confirmed here with the present methods; however, the relationship with lower airway disease status was not previously tested. In contrast to other β-diversity methods, UniFrac distances take into account the phylogenetic distance between ASVs. The weighted UniFrac distance additionally considers the abundance of ASVs, whereas the unweighted UniFrac only considers presence/absence (Lozupone et al., 2007). Interestingly, the unweighted UniFrac distance was not significant for any of these three variables. This indicates that similar patterns of presence/absence were evident but ASV abundance varied between patients with asthma/bronchiolitis and significant hearing loss.

Differential abundance analysis with DESeq2 (Love et al., 2014) indicated that four of the five most abundant genera, *Haemophilus, Turicella, Alloiococcus*, and *Moraxella*, were differentially abundant between patients with and without asthma/bronchiolitis (Figure 5). *Staphylococcus* was also significantly higher in patients with lower airway disease. These results highlight a potentially significant difference in the microbiome of COME patients with asthma or bronchiolitis. Both *Haemophilus* and *Moraxella* have been studied extensively in the asthma and bronchiolitis-related microbiome (Hilty et al., 2010; Vissing et al., 2013; Castro-Nallar et al., 2015; Pérez-Losada et al., 2015, 2017, 2018; Hasegawa et al., 2016; Mansbach et al., 2016); therefore, it is particularly interesting that these genera were found in greater abundance in asthma patients with COME. Early airway colonization with *Haemophilus* and *Moraxella* has been associated with asthma or bronchiolitis development later in childhood (Bisgaard et al., 2007; Vissing et al., 2013), and both have been found in higher abundance in patients with asthma or bronchiolitis (Hilty et al., 2010; Vissing et al., 2013; Castro-Nallar et al., 2015). *Staphylococcus*-dominated airway microbiomes has also been associated with an increased risk of bronchiolitis (Hasegawa et al., 2017). In contrast, *Turicella otiditis* and *Alloiococcus otitis* have been isolated almost exclusively from the human ear and have frequently been attributed to OM (Tano et al., 2008; von Graevenitz and Funke, 2014; Boers et al., 2018). The absence of these species from other parts of the upper respiratory tract has led to a debate about whether they are OM pathogens or part of commensal microbiota. However, it is striking that pathogens associated with asthma and bronchiolitis are increased in COME with lower airway disease, whereas pathogens typically associated with OM are decreased. These results suggest a potential link between the microbiome of the respiratory tract in children with lower airway disease and the microbiome associated with COME. Alternatively, the different microbial communities in patients with comorbid respiratory illnesses could be reflective of disease severity and increased pathogen load in the middle ear. In this study, significant hearing loss served as a proxy for symptom severity and was included in models for differential abundance in DESeq2. It is also possible that medications commonly prescribed for patients with lower airway disease such as corticosteroids could influence the microbiota present in both the lower and upper airways. Denner et al. (2016) showed that corticoid steroid use affects microbiome composition in the lower airways; however, it is not currently known whether this effect would extend to the upper airways. Future work should consider additional metrics of disease severity as well as medication use when evaluating this potential link between the middle ear and respiratory illnesses.

A large body of work suggests a link between atopic disease and OM, and particularly COME. These associated atopic diseases include allergic rhinitis (Alles et al., 2001; Kreiner-Moller et al., 2012; Roditi et al., 2016), eczema (MacIntyre et al., 2010), and asthma (Gamble et al., 1992; Eldeirawi et al., 2010; MacIntyre et al., 2010; Bjur et al., 2012), but the same associations are not always reproduced between studies, leading to significant debate about the nature of these associations (Zernotti et al., 2017). Gamble et al. (1992) hypothesized that COME in asthmatics is not an isolated disease, but instead represents an atopic disease affecting the mucociliary system of entire respiratory tract. Similarly, inflammation from bronchiolitis could result in impaired mucociliary clearance and make the middle ear more susceptible to pathogens. The interesting decrease in specific ear-trophic OM pathogens (*Alloiococcus* and *Turicella*) accompanied by an increase in asthma- and bronchiolitis-associated pathogens (*Haemophilus, Moraxella, Staphylococcus*) in asthma and bronchiolitis patients supports the theory that respiratory disease-associated COME may have a distinct etiology than COME in children without respiratory illnesses (Gamble et al., 1992; Zernotti et al., 2017). Although the directionality of this relationship is unknown, our findings show that COME in children with lower airway disease is characterized by a distinct microbiome, which may also explain the higher frequency of COME complications such as mucoid effusion and COME recurrence observed in asthma patients (Gamble et al., 1992). The relationship between these diseases and their respective microbiomes should be further investigated in future work.

## Conclusions

In this study, we performed a detailed characterization of the composition and function of the middle ear microbiome in children with COME. Using an ASV pipeline, we achieved greater resolution at the species and genus levels than using OTUs. Taxonomic profiles varied significantly between children, while microbiome function was remarkably similar across all patients. Furthermore, we identified significant differences in the middle ear microbiome associated with a lower airway disease diagnosis, including α- and β-diversity and relative abundance of *Haemophilus, Moraxella, Staphylococcus, Alloiococcus*, and *Turicella*. These results provide novel evidence linking the microbiome of respiratory illnesses with COME and warrant additional work on the link between these important childhood diseases.

## Data Availability Statement

Raw data files are available in the NCBI SRA under accession PRJNA555884.

## Ethics Statement

The studies involving human participants were reviewed and approved by Institutional Review Board, Children's National Health System. Written informed consent to participate in this study was provided by the participants' legal guardian/next of kin.

## Author Contributions

AK, EC-N, DP, and MP-L conceived and designed this study. AK analyzed the data and wrote the manuscript with contributions of all the authors. All authors read and approved this manuscript.

### Conflict of Interest

The authors declare that the research was conducted in the absence of any commercial or financial relationships that could be construed as a potential conflict of interest.

## Abbreviations

AOM: acute otitis media
ASV: amplicon sequence variant
COME: chronic otitis media with effusion
OM: otitis media.
