# Supplementary material for: Altered Middle Ear Microbiome in Children With Chronic Otitis Media With Effusion and Respiratory Illnesses
Source: Front Cell Infect Microbiol. 2019 Oct 4;9:339. doi: 10.3389/fcimb.2019.00339 (PMC6787523; doi:10.3389/fcimb.2019.00339)
Supplement: Supplementary file 1 [file Data_Sheet_1.PDF]

Supplementary Table 1: ASVs with mean relative abundance > 0.1% which were assigned at species level.

Species	Relative abundance (%) (Mean ± standard error)
Turicella otitidis	6.89 ± 2.18
Alloiococcus otitis	6.01 ± 1.66
Stenotrophomonas maltophilia	4.63 ± 1.44
Sarcina ventriculi	0.63 ± 0.63
Mesorhizobium loti	0.48 ± 0.22
Anaerococcus vaginalis	0.30 ± 0.29
Corynebacterium timonense	0.17 ± 0.14
Novosphingobium subterraneum	0.13 ± 0.06
Corynebacterium kroppenstedtii	0.12 ± 0.07

Supplementary Table 2: Prevalence (number of samples with nonzero abundance) and mean relative abundance for genera with >1% mean relative abundance.

Genus	Prevalence	Relative Abundance (Mean ± standard error)
Haemophilus	27/50	22.45 ± 5.26
Moraxella	26/50	13.31 ± 3.65
Turicella	26/50	9.03 ± 2.84
Achromobacter	39/50	6.64 ± 1.85
Alloiococcus	18/50	6.21 ± 1.72
Pseudomonas	38/50	6.12 ± 1.97
Pseudoflavitalea	27/50	5.31 ± 1.37
Stenotrophomonas	25/50	4.87 ± 1.47
Streptococcus	17/50	3.91 ± 2.09
Variovorax	31/50	2.93 ± 0.77
Staphylococcus	28/50	2.91 ± 1.38
Sphingomonas	33/50	1.53 ± 0.43
Brucella	22/50	1.14 ± 0.42
Mesorhizobium	30/50	1.02 ± 0.35
Gordonia	19/50	1.02 ± 0.40
